# COVID‐19 vaccine‐related new‐onset lichen planus

**DOI:** 10.1002/ccr3.5323

**Published:** 2022-02-02

**Authors:** Arefeh Babazadeh, Ronak Miladi, Mohammad Barary, Maria Shirvani, Soheil Ebrahimpour, Zeinab Aryanian, Zeinab Mohseni Afshar

**Affiliations:** ^1^ Infectious Diseases and Tropical Medicine Research Center Health Research Institute Babol University of Medical Sciences Babol Iran; ^2^ 48464 Clinical Research Development Center Imam Reza Hospital Kermanshah University of Medical Sciences Kermanshah Iran; ^3^ Student Research Committee Babol University of Medical Sciences Babol Iran; ^4^ 114456 Students' Scientific Research Center (SSRC) Tehran University of Medical Sciences Tehran Iran; ^5^ 114456 Autoimmune Bullous Diseases Research Center Tehran University of Medical Sciences Tehran Iran; ^6^ Department of Dermatology School of Medicine Babol University of Medical Sciences Babol Iran

**Keywords:** COVID‐19, dermatology, lichen planus, SARS‐CoV‐2

## Abstract

Coronavirus disease 2019 (COVID‐19) vaccines significantly impacted world health and well‐being. However, various adverse events have been observed following severe acute respiratory syndrome coronavirus 2 (SARS‐CoV‐2) vaccination. Cutaneous reactions have been prevalent following many vaccines, including COVID‐19 vaccines. Here, we present a case of new‐onset lichen planus in a patient who received the COVID‐19 vaccine at the same time as being infected with SARS‐CoV‐2. A 52‐year‐old woman presented to the clinic with extensive pruritic skin lesions. The eruptions had appeared a week after her second dose of the Sinopharm COVID‐19 vaccine. She mentioned a history of SARS‐CoV‐2 infection approximately 10 days following the first dose of her vaccine, causing a 1‐month delay in getting the second dose. Her past medical history was not significant. On examination, erythematous and squamous papules were demonstrated predominantly on the extremities, including inguinal and axillary folds. Moreover, desquamation of the lips was visible, and buccal lesions were also found. After consultation with a dermatologist, a skin biopsy was indicated for the patient, but she refused to undergo the procedure. Therefore, considering the typical appearance of the eruptions, lichen planus was suspected, for which she was treated with oral antihistamines and topical corticosteroids.

## Abstract 2

Dermatologists should be aware of the probability of new‐onset or exacerbated mucosal skin disorders due to the vast range of cutaneous adverse events following COVID‐19 vaccination and actively monitor susceptible patients.

## 1 INTRODUCTION

Coronavirus disease 2019 (COVID‐19) vaccines significantly impacted world health and well‐being. However, various adverse events have been observed following severe acute respiratory syndrome coronavirus 2 (SARS‐CoV‐2) vaccination. Cutaneous reactions have been prevalent following many vaccines, including COVID‐19 vaccines. The SARS‐CoV‐2 infection per se has led to different dermatologic manifestations. Psoriasis and seborrheic dermatitis are among the most common long‐lasting dermatologic disorders triggered or flared up following this viral infection or related vaccines.^1^, ^2^ Nonetheless, lichen planus has been reported less commonly after SARS‐CoV‐2 infection or COVID‐19 vaccination, necessitating its careful discussion.^3^, ^4^ Here, we present a case of new‐onset lichen planus in a patient who received the COVID‐19 vaccine at the same time as being infected with SARS‐CoV‐2.

## 2 CASE PRESENTATION

**FIGURE 1:**
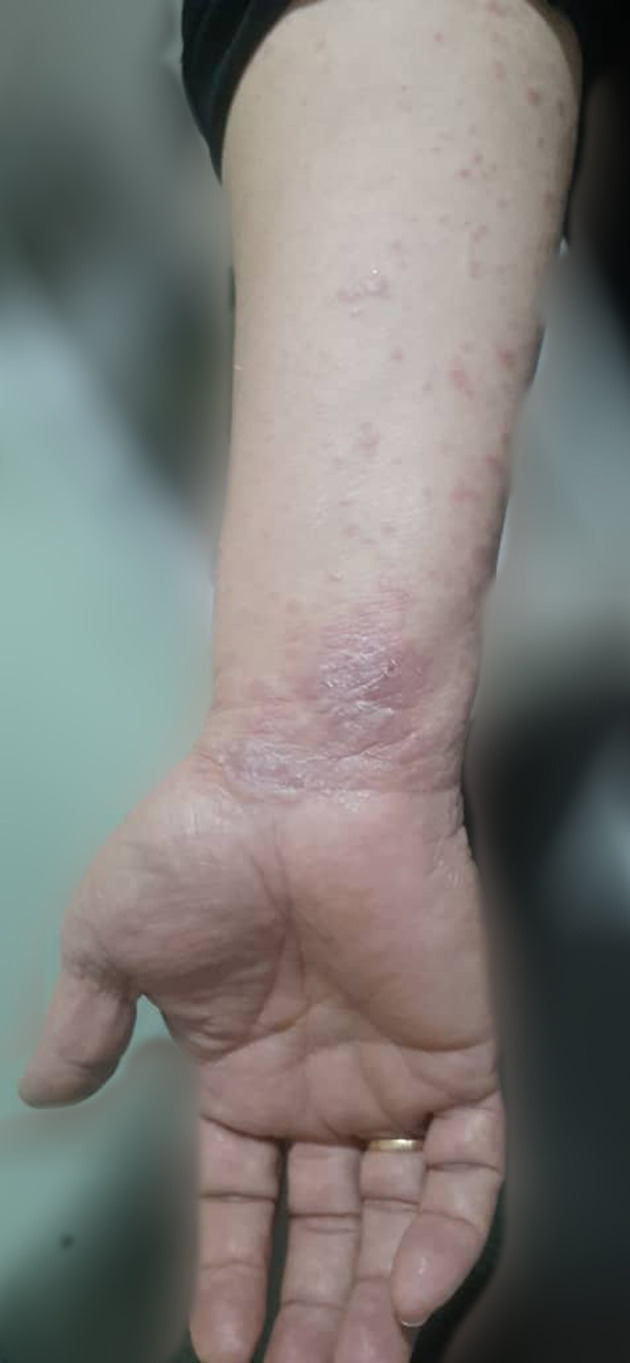
Lichen planus lesions of the patient's forearm

**FIGURE 2:**
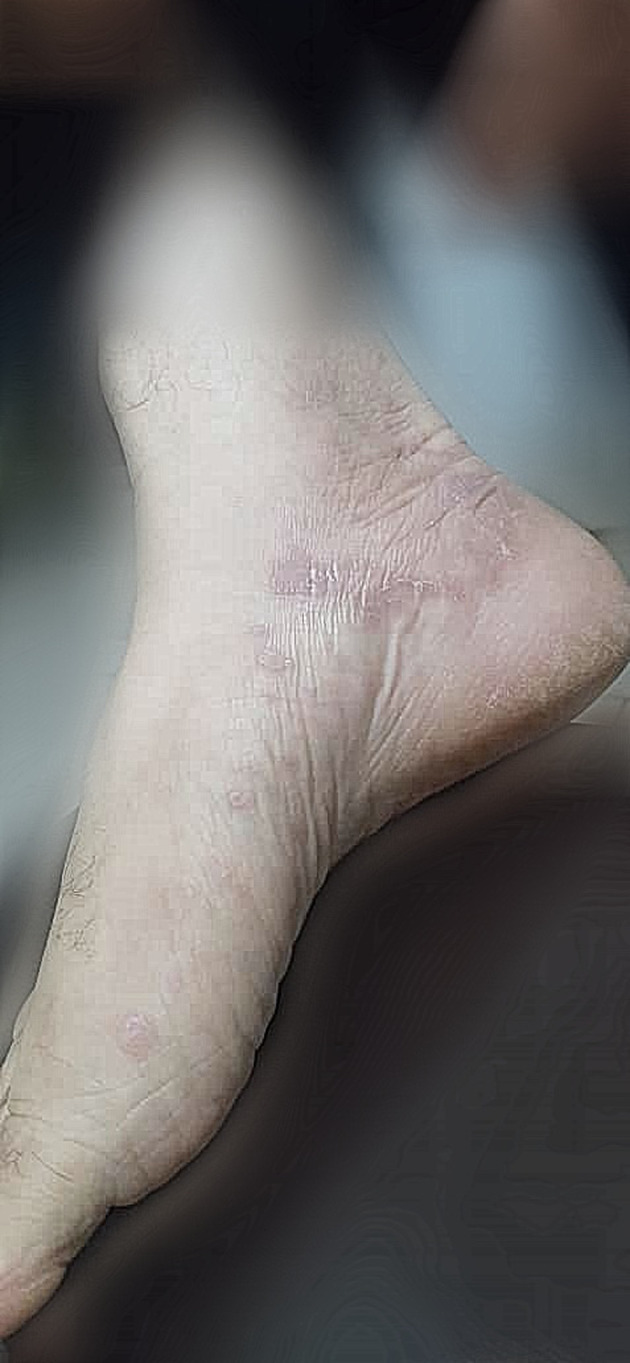
Lichen planus lesions of the patient's leg and ankle

A 52‐year‐old woman presented with extensive pruritic skin lesions was referred to the infectious diseases clinic. The eruptions had appeared a week after her second dose of the Sinopharm (BBIBP‐CorV) COVID‐19 vaccine. She mentioned a history of SARS‐CoV‐2 infection approximately 10 days following the first dose of her vaccine, causing a 1‐month delay in getting the second dose. Her past medical history was not significant. On examination, erythematous and squamous papules were demonstrated predominantly on the extremities, including inguinal and axillary folds (Figures 1 and 2). Moreover, desquamation of the lips was visible, and buccal lesions were also found. After consultation with a dermatologist, a skin biopsy was indicated for the patient, but she refused to undergo the procedure. Therefore, considering the typical appearance of the eruptions, lichen planus was suspected, for which she was treated with oral antihistamines and topical corticosteroids. She was then visited during her follow‐up with evidence of a favorable response to the treatment.

## 3 DISCUSSION

Unspecific skin complications, such as injection site induration, urticaria, and maculopapular rash, have frequently been observed following various vaccines, including SARS‐CoV‐2 vaccines, often transient and self‐limited.^5^ However, dermatologic disorders, such as psoriasis and lichen planus, have been reported less commonly, but their durability and complex treatment pose significant challenges for the patients.

Lichen planus is a chronic, inflammatory, autoimmune dermatologic disorder that involves the skin and mucosal membranes. Cutaneous lesions usually present with planar, purple, polygonal, pruritic, papules, and plaques, known as the six Ps. Mucosal surfaces that are commonly affected include oral and genital mucosa. Moreover, skin appendages such as nails and scalp hair are sometimes engaged.^6^ Lichen planus frequently presents acutely and involves the flexor surfaces of the upper and lower extremities.^7^ Its cutaneous lesions are often covered by Wickham striae, manifested as reticular purple pruritic papules and plaques in flexural areas and oral lesions on the lips and buccal mucosae. Our patient had typical cutaneous and mucosal involvement, but her nails and hair were spared.

Lichen planus had been known to be triggered by several factors, including systemic comorbidities (e.g., hypertension, diabetes mellitus, and chronic liver disease), medications (such as beta‐blockers, nonsteroidal anti‐inflammatory drugs, antimalarials, diuretics, oral hypoglycemics, and penicillamine), infections (including hepatitis B and C viruses, and *Helicobacter pylori*), tobacco chewing, and anxiety.^8^, ^9^, ^10^, ^11^, ^12^ Nonetheless, based on the previous studies, SARS‐CoV‐2 infection can be another precipitating factor for this condition.^13^, ^14^, ^15^, ^16^ Moreover, SARS‐CoV‐2 vaccines have been the other factors that could induce this disease. Previously, hepatitis B vaccine has been abundantly reported to cause lichen planus, and other vaccines, such as tetanus–diphtheria–acellular pertussis (Tdap), measles–mumps–rubella (MMR), rabies, and influenza vaccines, had also been reported to trigger this cutaneous disorder less frequently.^17^, ^18^, ^19^, ^20^, ^21^, ^22^ However, some reports of new‐onset lichen planus occurring following COVID‐19 vaccines.^3^, ^23^, ^24^ Furthermore, exacerbation of the pre‐existing lichen planus has also been observed after COVID vaccines.^23^ The pathophysiologic mechanism might be the T_h1_ response that is elicited by the vaccines, which in turn leads to the elevation of interleukin‐2 (IL‐2), tumor necrosis factor‐α (TNF‐α), and interferon‐γ (IFN‐γ) levels, and therefore, lichen planus induction.^25^ Our patient did not point out any of the risks mentioned above, and her past medical and medication history was not significant. Antimalarial agents are the most critical medications involved in the rising up or exacerbation of lichen planus, but our patient had not received hydroxychloroquine to treat her SARS‐CoV‐2 infection. In addition, her laboratory tests showed a negative serology for hepatitis B, hepatitis C, and HIV. The result of the SARS‐CoV‐2 RT‐PCR test was also negative.

Both SARS‐CoV‐2 infection and its vaccines can serve as triggering factors of cutaneous adverse reactions such as lichen planus. Since our patient got infected with the virus in the meantime between the two doses of the vaccines, it is difficult to determine whether she developed this dermatologic disease due to SARS‐CoV‐2 infection or as a consequence of its vaccines. Reports of lichen planus onset following SARS‐CoV‐2 infection have demonstrated that the cases had occurred up to 1 month after the infection.^4^, ^26^ On the other hand, the few reports of lichen planus following SARS‐CoV‐2 vaccination had occurred about 1–2 weeks after receiving the vaccines.^3^, ^27^, ^28^ In a repeated and more thorough history taking, the patient recalled some faint and scares lesions arising a few days following her first vaccine dose on her both ankles, which faded away, and recurred soon after the second vaccine dose with a more robust appearance, so as obliging her to seek physician visits. Therefore, this patient's onset of lichen planus can almost definitely be due to COVID vaccination because her first bout of cutaneous reactions had appeared prior to getting infected with SARS‐CoV‐2. Cutaneous biopsy is the best way to confirm the diagnosis of a dermatologic disorder, including lichen planus^29^; if punch biopsy had been done, “saw‐tooth” pattern of epidermal hyperplasia with wedge‐shaped hypergranulosis, hyperparakeratosis, and vacuolar alteration of the basal layer of the epidermis, with predominant T cells infiltration at the dermal–epidermal junction would be the characteristic finding^30^; however, our patient refused to undergo biopsy. Therefore, we considered the diagnosis based upon the typical clinical manifestations. High‐potency topical corticosteroids are the first‐line medications utilized for lichen planus treatment.^31^ However, if no favorable response is demonstrated, topical calcineurin inhibitors, such as tacrolimus and pimecrolimus, are usually applied as second‐line therapies, particularly for oral and genital lesions.^32^ Systemic corticosteroids are another choice for severe, widespread mucosal lesions.^33^ Lichen planus may resolve spontaneously or with the treatments mentioned above within 1–2 years; however, recurrences occur prevalently, especially for mucosal lichen planus, which may persist for the lifelong duration despite therapy.^34^ Our patient's cutaneous lesions responded favorably to topical calcipotriol and triamcinolone in addition to systemic antihistamines; nonetheless, her buccal mucosae and lip involvement had not subsided. Therefore, she was started on oral prednisone.

## 4 CONCLUSION

Although COVID‐19 vaccines and their adverse events are new scopes in medicine, dermatologists should be aware of the probability of new‐onset or exacerbated mucosal skin disorders due to the vast range of cutaneous adverse events following COVID‐19 vaccination and actively monitor susceptible patients.

## CONFLICT OF INTEREST

The authors declare that they have no conflict of interest.

## AUTHOR CONTRIBUTIONS

The case was diagnosed and followed up by AB and ZMA. RM and MB conceived and planned the case report. MS, SE, and ZA wrote the manuscript, and MB edited the first draft and provided substantial revision. The final version was read, corrected, and approved by all authors. All co‐authors take full responsibility for the integrity of the case study and literature review.

## ETHICAL APPROVAL

Not applicable.

## CONSENT

Written informed consent was obtained from the patient for publication of the current case report.

## Data Availability

The data that support the findings of this study are available from the corresponding author upon reasonable request.
